# Reproducibility of T1 mapping 11-heart beat MOLLI Sequence

**DOI:** 10.1186/1532-429X-17-S1-W26

**Published:** 2015-02-03

**Authors:** Vassilis Vassiliou, Ee Ling Heng, Pranev Sharma, Evangelia Nyktari, Claire E Raphael, Calvin W Chin, Peter Drivas, Gillian C Smith, Karen Symmonds, George Lathra Mathew, Ricardo Wage, Aamir Ali, Andreas Greiser, Francisco Alpendurada, Marc R Dweck, Dudley J Pennell, Peter Gatehouse, Sanjay K Prasad

**Affiliations:** 1 CMR, Royal Brompton Hospital, London, UK; 2 National Heart and Lung Institute, Imperial College London, London, UK; 3 BHF Centre for Cardiovascular Science, Edinburgh, UK; 4 Siemens, Germany, Germany

## Background

T1 mapping measuring the longitudinal relaxation time constant is increasingly used clinically for quantification of interstitial myocardial fibrosis, with higher myocardial T1 and ECV associated with adverse prognosis (Wong 2012). A working group consensus statement (Moon 2013) has recommended amongst other:

1) Use of native T1 myocardium and ECV only, avoiding partition coefficient (λ)

2) Use of gadolinium-based contrast agents (GBCA) for ECV calculation routinely and post-gad T1 maps taken at least 15 minutes following administration

3) Use of at least 2 different slice orientations to add diagnostic confidence

We sought to investigate the reproducibility of an 11-cycle 5(3)3 MOLLI sequence (Siemens prototype WIP448B) and furthermore wished to investigate whether repeating the MOLLI acquisition on one slice orientation twice could increase precision.

## Methods

**Figure 1:**
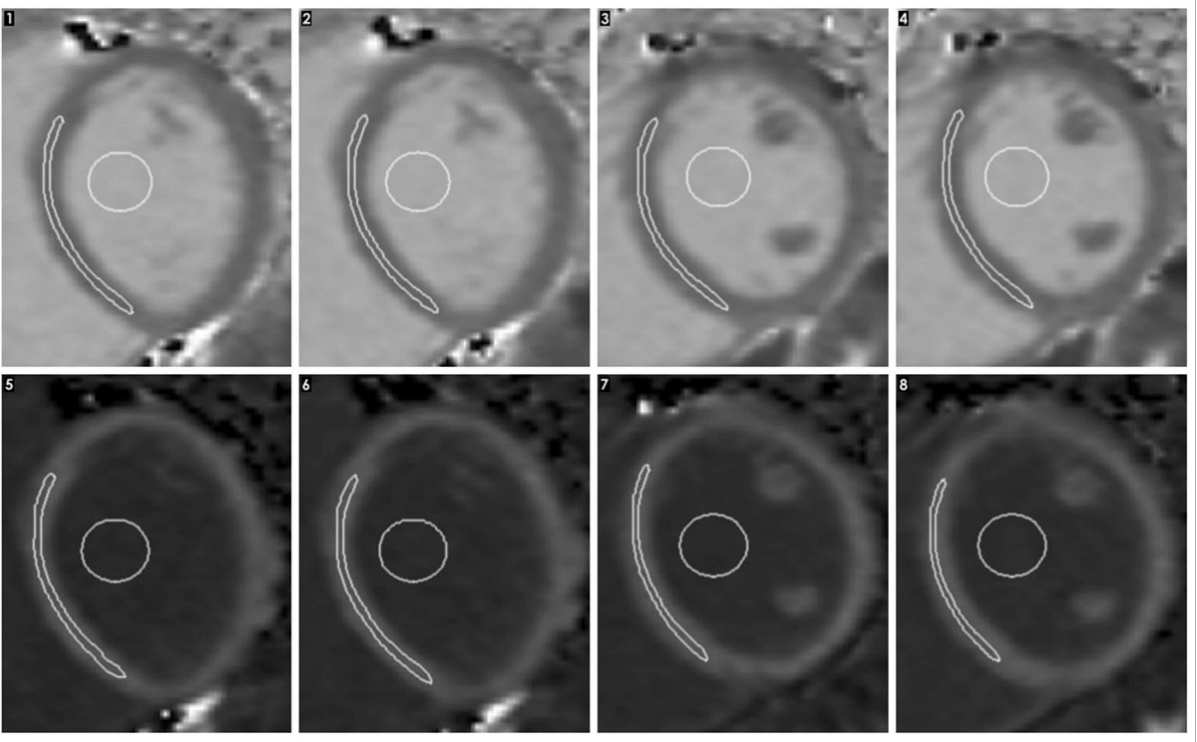
shows native T1 maps (top series numbers 1-4) and post gad (bottom series numbers 5-8). One basal and one mid level were selected and the acquisition repeated twice both before and after the administration of gad. Regions of interest (ROI) were drawn in the myocardium and blood. The scan was repeated using the same protocol within 60 days. To mimic routine clinical practice, the staff undertaking the second scan were not aware of the slice position used for acquisition during the first scan. There were no significant differences in heart rate between the two scans.To mimic routine clinical practice, the staff undertaking the second scan were not aware of the slice position used for acquisition during the first scan. There were no significant differences in heart rate between the two scans.

15 healthy volunteers (31±5 years, 8 males) with no known medical conditions, on no medication, underwent CMR scans (Avanto, Siemens, 1.5T) on two separate attendances with an 11 heart beat MOLLI 5(3)3. Following frequency adjustment, native T1 maps were obtained twice on a basal and a mid-ventricular slice respectively. 15 mins following GBCA administration (0.1 mmol/kg, Gadobutrol, Bayer, Germany) both image planes were acquired again twice. Pixel-wise T1 maps were analyzed offline by 2 independent blinded operators. A Region Of Interest was manually drawn in the septum for myocardium and blood in native and post gad images as shown in fig 1.

## Results

Intrastudy and interstudy intraclass coefficient (ICC) was assessed for various models (model A-F) combining an increasing number of basal and mid level acquisitions as shown in table 1. Intrastudy ICC was excellent for native T1 myocardium and ECV (0.94 and 0.86), but not for λ (0.54). Interstudy ICC for native T1 was very good for all the models [highest model E (0.90), lowest model B (0.77)]. Interstudy ICC for λ was overall poor [highest model F (0.79), lowest model B (0.70)]. For ECV there was excellent interstudy ICC when at least 2 native and 2 post-gad slices were included, best model F ( 0.93; with 1 basal and 1 mid level acquisition repeated, total 4 acquisitions native and 4 post gad). Interobserver reproducibility was excellent with intra and inter scan reproducibility across all models for T1, λ and ECV >0.90 (highest model F ECV ICC 1.00, lowest model E λ ICC 0.94).

**Table 1:** showing the 6 models and their corresponding ICCs. The average ICC of the two observers was used. Native T1 and ECV have excellent reproducibility, particularly when model F is used where one mid level and one basal level are selected, and the acquisitions repeated twice both before and after gad, allowing for the generation of 4 native (2 basal and 2 mid) and 4 post gad maps. Given the increased reproducibility, at the only expense of two extra short breathholds, repeating each slice twice could be routinely considered in all scans where T1 maps are undertaken.

Intrastudy and intersudy reproducibility								
					Native T1 myocardium	Post gad T1 myocardium	λ	ECV

INTRASTUDY	Basal Level	Repeated	Mid Level	Repeated	ICC	ICC	ICC	ICC

Model A			x		0.96	0.83	0.79	0.90

Model B	x				0.95	0.87	0.72	0.91

Model C	x		x		0.94	0.87	0.54	0.86

INTERSTUDY	Basal Level	Repeated	Mid Level	Repeated	ICC	ICC	ICC	ICC

Model A			x		0.90	0.71	0.50	0.82

Model B	x				0.77	0.70	0.39	0.77

Model C			x	x	0.84	0.81	0.73	0.90

Model D	x	x			0.80	0.76	0.67	0.89

Model E	x		x		0.90	0.71	0.54	0.86

Model F	x	x	x	x	0.88	0.79	0.80	0.93

## Conclusions

Interpreted together these results suggest an 11 heart beat MOLLI prototype sequence can have high intra and inter scan and interobserver reproducibility. ECV shows the best reproducibility especially when images of one basal and one mid level are repeated twice (model F) and that T1 native myocardium reproducibility is excellent independently of model used. We would therefore recommend that if only 2 levels are acquired with the MOLLI sequence a repeat acquisition should be considered at each slice, to increase precision and reproducibility.

## Funding

NIHR BRU, Royal Brompton Hospital, London.

